# Investigating the Potential of Essential Oils from *Citrus reticulata* Leaves in Mitigating Memory Decline and Oxidative Stress in the Scopolamine-Treated Zebrafish Model

**DOI:** 10.3390/plants13121648

**Published:** 2024-06-14

**Authors:** Ion Brinza, Razvan Stefan Boiangiu, Iasmina Honceriu, Ahmed M. Abd-Alkhalek, Omayma A. Eldahshan, Gabriela Dumitru, Lucian Hritcu, Elena Todirascu-Ciornea

**Affiliations:** 1 Department of Biology, Faculty of Biology, Alexandru Ioan Cuza University of Iasi, 700506 Iasi, Romania; ion.brinza@student.uaic.ro (I.B.); razvan.boiangiu@uaic.ro (R.S.B.); iasmina.honceriu@student.uaic.ro (I.H.); ciornea@uaic.ro (E.T.-C.); 2 Faculty of Medicine (for Boys), Al Azhar University, Cairo 11884, Egypt; ahmedahmed1.stu.1@azhar.edu.eg; 3 Department of Pharmacognosy, Faculty of Pharmacy, Ain Shams University, Abbassia, Cairo 11566, Egypt; oeldahshan@pharma.asu.edu.eg; 4 Center of Drug Discovery Research and Development, Ain Shams University, Cairo 11566, Egypt

**Keywords:** anxiety, Alzheimer’s disease, *Danio rerio*, memory, oxidative stress, pharmacokinetics, petitgrain essential oil, scopolamine

## Abstract

Petitgrain essential oil (PGEO) is derived from the water distillation process on mandarin (*Citrus reticulata*) leaves. The chemical constituents of PGEO were analyzed by gas chromatography/mass spectrometry (GC/MS) method which revealed the presence of six compounds (100%). The major peaks were for methyl-N-methyl anthranilate (89.93%) and γ-terpinene (6.25%). Over 19 days, zebrafish (Tubingen strain) received PGEO (25, 150, and 300 μL/L) before induction of cognitive impairment with scopolamine immersion (SCOP, 100 μM). Anxiety-like behavior and memory of the zebrafish were assessed by a novel tank diving test (NTT), Y-maze test, and novel object recognition test (NOR). Additionally, the activity of acetylcholinesterase (AChE) and the extent of the brain’s oxidative stress were explored. In conjunction, in silico forecasts were used to determine the pharmacokinetic properties of the principal compounds discovered in PGEO, employing platforms such as SwissADME, Molininspiration, and pKCSM. The findings provided evidence that PGEO possesses the capability to enhance memory by AChE inhibition, alleviate SCOP-induced anxiety during behavioral tasks, and diminish brain oxidative stress.

## 1. Introduction

Cognitive decline is a prevalent problem in the elderly population and is a prominent factor in mortality rates. World Health Organization projections suggest that more than 55 million people globally experience severe cognitive impairment, also known as dementia. Dementia significantly impairs the well-being of patients and their families, placing significant strain on health systems, communities, and the global financial landscape. With aging demographics on a global scale and the extended lifespan of people affected by dementia, it is anticipated that the repercussions of this condition will increase. Despite various potential contributors to the onset of dementia, Alzheimer’s disease (AD) is recognized as the main culprit [1]. AD is a complex neurodegenerative condition, characterized by the presence of distinctive pathological changes at the brain level. These changes include extracellular plaques containing β-amyloid (Aβ) and intracellular neurofibrillary tangles (NFTs) containing the hyperphosphorylated tau protein [2]. Important consequences of NFTs are determined by the mislocalization of the tau protein inside nerve cells. The tau protein is normally present in neuronal axons, where it helps maintain their structure and function. However, when hyperphosphorylated, the tau protein mislocalizes to other parts of the nerve cell, including dendritic spines, thereby affecting the ability of neurons to communicate effectively with each other [3]. Several studies have revealed the extensive degeneration of cholinergic neurons in the basal nucleus of the brain in patients with AD, thus suggesting a possible involvement of the cholinergic system in the cognitive deficit observed. However, recent research suggests that the various mechanisms proposed for the pathophysiology of AD may not be exclusive but may interact with each other [4]. For example, it has been found that exposure of cholinergic neurons to Aβ can induce cytotoxicity, contributing to nerve cell damage. In addition, activation of these neurons has been associated with changes in Aβ processing and tau protein phosphorylation, both of which are hallmarks of AD [5]. Also, research has shown that acetylcholinesterase (AChE), an enzyme that breaks down acetylcholine (ACh), the main neurotransmitter of the cholinergic system, and presenilin 1, which is part of the γ-secretase enzyme complex involved in the production of Aβ, interact and can influence each other. This interaction may modulate the expression and activity of these enzymes, impacting cholinergic homeostasis and Aβ production [6]. In addition, the neurogenesis and cognitive decline observed in AD pathogenesis are also associated with oxidative damage induced by increased oxidative stress, which involves the oxidation of membrane proteins, lipids, and carbohydrates, especially in the neuronal membrane [7].

AD usually manifests as difficulties in semantic short-term memory but evolves to affect many other higher cognitive functions. During disease progression, multiple other cognitive and behavioral disturbances are common and variable. These may include but are not limited to anxiety, depression, apathy, irritability, euphoria, agitation, loss of inhibition, psychosis, aggression, unusual motor behavior, sleep disturbance, and eating disorders. The average survival time for people with AD is about 7 years [8].

Because of its similarity to human neural networks, the zebrafish has become a promising study model for AD research. This model has been well validated by research that has analyzed neural pathways analogous to those in the human brain. Cholinergic, glutamatergic, and GABAergic pathways, which play an important role in zebrafish behavior, are well defined and have been studied in detail. Several behavioral models are used in adult zebrafish to examine different facets of cognitive impairments, including spatial memory, associative memory, anxiety, and other phenomena seen in AD. The use of zebrafish as a research model allows for the evaluation of the effectiveness of therapeutic interventions more conveniently and efficiently, compared to the use of alternative animal models such as mice or rats. The use of zebrafish addresses certain limitations of mammalian models previously used in research, such as high expense, prolonged assessment times, and complexity of brain imaging. Thus, this model provides a simpler and more accessible platform for investigating AD and other neurological disorders, thereby streamlining the process of developing and evaluating potential therapeutic interventions [9,10].

The *Citrus* genus is known for its contribution to medicinal activities [11,12,13,14]. Petitgrain essential oil (PGEO) is derived from the leaves and green twigs of the mandarin plant (*Citrus reticulata*).

PGEO is used to calm the central nervous system and relieve stress. Also, it exerts a tonic effect, affects flatulence, constipation, diarrhea and increases skin circulation, reduces the retention of fluids, and prevents stretch marks [15]. Also, it has a broad-spectrum antibacterial and antifungal activity by inhibiting their growth [16,17,18]. Our previous study on a mixture of mandarin petitgrain and marjoram oils revealed a synergistic effect on *Helicobacter pylori* at a minimum inhibitory concentration (MIC) of 1.95 µg/mL (the same as clarithromycin, at the same concentration) [19]. In addition, mandarin leaf essential oil showed potent antioxidants, anticancer (HL-60 and NB4 cell lines), and antioxidant activity through the radical scavenging effect [20].

This study aimed to identify the neuroprotective mechanisms of PGEO and evaluate its potential in the treatment and prevention of dementia-related conditions. For this, a zebrafish model was used with cholinergic deficiency induced by scopolamine (SCOP). The results of this study could provide new insights into the therapeutic use of essential oils in the treatment of dementia and other neurological conditions.

## 2. Results and Discussion

### 2.1. Chemical Composition of PGEO

Six compounds accounting for 100% of the total PGEO were identified. The results of the gas chromatography/mass spectrometry (GC/MS) analysis of the major components of PGEO are presented in Table 1.

The major compounds were methyl-N-methyl anthranilate (89.93%) and *γ*-terpinene (6.25%), respectively. Our results agree with those reported by other authors. From a quantitative perspective, some of the most abundant compounds were *γ*-terpinene, sometimes reaching values above 15% [20,21], and the aromatic compound methyl *N*-methyl anthranilate (0.7–0.1%) [22].

### 2.2. Highlighting the Biomedical Alert Structures of PGEO Main Biocompounds

**Figure 1:**
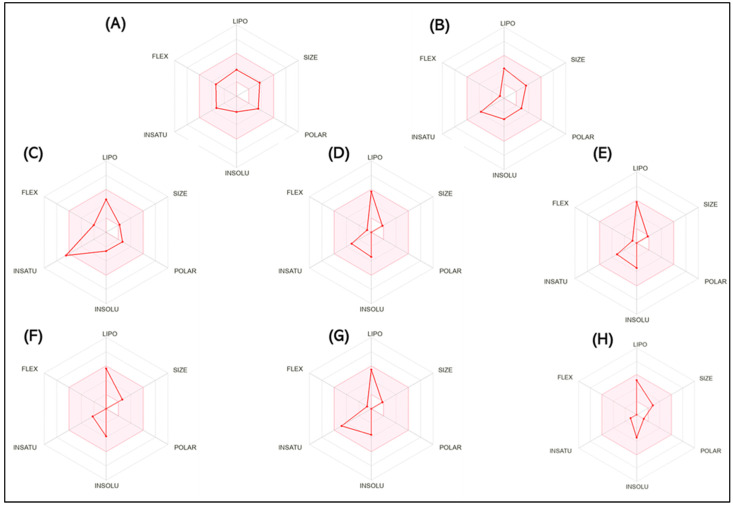
The bioavailability radar indicates the optimal range for each property. For lipophilicity, the pink area corresponds to the octanol/water partition coefficient (LogP) between −0.7 and +5.0. For size, the optimal range is given by a molecular weight between 150 and 500 g/mol. In terms of polarity, the total polar surface area (TPSA) ranges from 20 to 130 Å. For solubility, the pink area is defined by LogS values < 6. For saturation, the fraction of carbon atoms in sp3 hybridization is greater than 0.25. Finally, for flexibility, a maximum number of nine rotating links is allowed. (**A**) Scopolamine (SCOP), (**B**) Galantamine (GAL), (**C**) Methyl-N-methyl anthranilate, (**D**) γ-Terpinene, (**E**) Limonene, (**F**) β-Caryophyllene, (**G**) p-Cymene, and (**H**) Caryophyllene oxides can be dissolved orally.

In Figure 1, the bioavailability radar is presented to highlight the characteristics of the analyzed substances, with special emphasis on six physio-chemical attributes: lipophilicity, size, polarity, solubility, flexibility, and saturation. Our conclusions suggest that all eight substances investigated in the analysis (SCOP, GAL, methyl-N-methyl anthranilate, γ-terpinene, limonene, β-caryophyllene, p-cymene, and caryophyllene oxide) can be dissolved orally.

According to studies by Xiong et al. [23], compounds with pharmaceutical potential should fall within certain ranges for their physicochemical properties. These details are summarized in Table 2. Thus, the molecular weight of the compounds should be between 100 and 600 g/mol. The optimum range for the number of hydrogen bond acceptors (nH) is between 0 and 12, and for the number of hydrogen bond donors (NHd), the optimum range is between 0 and 6. For the total polar surface area (TPSA), the value should be between 20 and 130 Å, as well as for water solubility (LogS). Also, the octanol/water partition coefficient (LogP) should be between −0.7 and +5.0. To respecify the criteria, the fraction of carbon atoms in sp3 hybridization (Csp3) must not be less than 0.25 and the number of rotatable bonds (e) must not exceed nine. These recommendations provide a framework useful for the selection and evaluation of compounds with pharmaceutical potential.

### 2.3. The Similarities between the Pharmacokinetic Properties of the Compounds

Five complementary drug-likeness rules were chosen, namely Lipinski’s Rule [24], Weber’s Rule [25], Ghose’s Rule [26], and Egan’s Rule [27], and the bioavailability score was also computed to study the medicinal chemical properties. These rules have been developed over time in the drug development process of reputable pharmaceutical companies.

The drug-likeness and medicinal chemistry properties of the selected compounds are presented in Table 3. Based on the obtained results, the SCOP and GAL compounds successfully passed all five filters (Lipinski, Ghose, Veber, Egan, and Muegge) without any violations. In contrast, the other compounds primarily found in PGEO showed at least one violation in these drug-like filters, including Methyl-N-methyl anthranilate (MW < 200), γ-terpinene (MW < 160, Heteroatoms < 2), limonene (MW < 160, Heteroatoms < 2), β-caryophyllene (MW < 200), p-cymene (MW < 160, Heteroatoms < 2), and și caryophyllene oxide (MW < 160, Heteroatoms < 2). The Abbot bioavailability score, calculated for all compounds, indicated that they fall into the 55% probability class, as shown in Table 3.

### 2.4. Evaluation of the Pharmacological Properties of PGEO

Oral administration is the most widespread and convenient method of drug administration, especially from the patient’s perspective. However, the pharmacokinetics of orally administered drugs depend on the rate of their absorption through the intestinal wall before reaching systemic circulation [28]. According to Table 3, the predictive results of the pharmacogenomic profile show that both SCOP and GAL, as well as methyl-N-methyl anthranilate, γ-terpinene, limonene, β-caryophyllene, p-cymene, and caryophyllene oxide, show high intestinal absorption. Previously, SCOP uptake was investigated using the isolated loop technique in a study of conscious rats. Following the administration of varying doses (0.5 mg, 1.0 mg, 2.0 mg, and 5.0 mg) in loops, it was found that after 3 h, an average of 36% of each injected dose was absorbed. A close correlation was also observed between the mean values of percent absorption in all groups [29]. Cannabinoids such as β-caryophyllene and caryophyllene oxide produce several effects on the gastrointestinal tract, which include regulating food intake, altering visceral sensation, reducing inflammation, and adjusting intestinal permeability. This permeability is mainly associated with mucus secretion and proteins that form tight junctions, with studies showing changes in mucus layer thickness and claudin-1 protein expression in the colon [30].

In terms of skin permeability, SCOP and GAL have low permeability, while methyl-N-methyl anthranilate, γ-terpinene, limonene, β-caryophyllene, p-cymene, and caryophyllene oxide can cross the skin epithelium easily.

Common methods of assessing the volume of distribution in either humans or animals include the calculation of the steady-state volume of distribution (VDss), the determination of VD β from the clearance and elimination rate constant (terminal phase volume), or the ratio of dose and the concentration at time 0 (VDc), where C0 is the extrapolated concentration at time 0 after the intravenous administration of a dose. In each of these cases, these values do not reflect the actual distribution of the compound (such as distribution between tissues and plasma), nor do they provide insight into the mechanism of distribution. Rather, they are constants of proportionality that indicate the tendency of a compound to leave the plasma (or blood) compartment and reach the tissues, without indicating a specific distribution among the different tissues [31]. pCKSM predictions [32] have indicated that methyl-N-methyl anthranilate and p-cymene present a low VDss, while the other analyzed substances have the potential to be rapidly distributed in the body after administration (according to Table 4). In addition, according to the data presented in the table, all substances can cross the blood–brain barrier (BBB) and reach the brain. At the same time, methyl-N-methyl anthranilate and caryophyllene oxide have the potential to penetrate the central nervous system (CNS), while the other analyzed compounds have a lower potential in this regard (according to Table 3). The brain, being the most important organ in our body, requires a unique environment for optimal functioning. The BBB is located at the microvascular interface of the brain, between blood and brain tissues, and plays an essential role in maintaining the optimal composition of the extracellular environment in the CNS, necessary to support neuronal functions. The distinct compositional differences between brain interstitial fluid and blood plasma underscore the importance of the BBB in preventing unwanted and potentially harmful substances from entering the brain environment [33,34]. Multiple studies show that SCOP [35,36] and GAL [37] can cross the BBB and reach the CNS.

**Figure 2:**
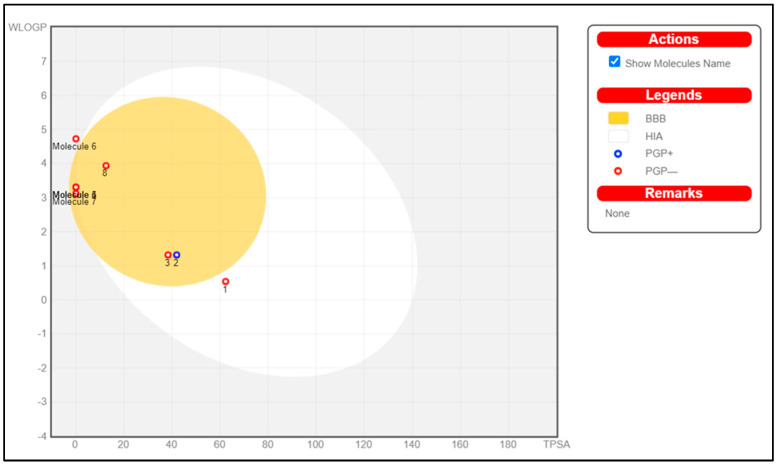
Instinctive assessment of passive gastrointestinal absorption (HIA) of (**1**) SCOP, (**2**) GAL, (**3**) Methyl-N-methyl anthranilate, (**4**) γ-Terpinene, (**5**) Limonene, (**6**) β-Caryophyllene, (**7**) p-Cymene, and (**8**) Caryophyllene oxide and brain penetration (BBB) based on molecular positioning in the WLOGP versus TPSA referential. The color reformation on the chart represents different aspects of drug absorption and penetration. The white region indicates a high probability of passive absorption in the gastrointestinal tract, while the yellow region indicates an increased probability of brain penetration. The two regions are not mutually exclusive. Dots are also colored blue if predicted to be actively effluxed by P-gp (PGP+) and red if predicted to be a non-substrate of P-gp (PGP−).

In addition, to ensure that at least one of the analyzed compounds from PGEO could penetrate the BBB and reach the CNS, we developed with the help of SwissADME [38] an ovular classification diagram showing a yolk and egg white as physicochemical domains associated with BBB penetration and human intestinal absorption, respectively (Figure 2). The outer gray region indicates molecules with limited uptake and penetration into the brain [38,39].

Thus, as can be seen in Figure 2, SCOP has the highest probability of human intestinal absorption, but a low probability of crossing the BBB. β-Caryophyllene has the weakest absorption and a low probability of crossing the BBB. GAL is predicted to enter the BBB through a passive process (shown in the yolk diagram) but is subsequently cleared from the brain (blue dot indication). Methyl-N-methyl anthranilate, γ-terpinene, limonene, p-cymene, and caryophyllene oxide enter the brain (indicated in the yolk diagram) and are not subject to active expulsion (indicated by a red dot). Sanchez-Martinez et al. [40] have previously shown that mono- and sesquiterpenoid hydrocarbons from industrial orange juice demonstrate a high ability during in vitro studies to cross the BBB. Satou et al. [41] showed that limonene shows a maximum concentration in mouse brains 90 min after inhalation.

Except for GAL, no other compound is a CYP3A4 substrate, and caryophyllene oxide is the only compound capable of inhibiting CYP1A2. Also, except for GAL, none of the analyzed compounds have the potential to be an OCT2 substrate, which indicates that GAL can penetrate tissues much faster than the other compounds in this study. In addition, except for GAL, no other compound shows liver toxicity. Beijsterveldt et al. [42] showed that after intravenous administration of GAL at concentrations of 1.25 and 2.5 mg/kg in rats, plasma levels exhibited a bi- or tri-phasic decline, with an elimination half-life ranging from 3.5 h in males to 5.1 h in females. The plasma clearance and volume of distribution were constant, indicating uniform distribution in the body. After oral administration, GAL is rapidly absorbed with significant absolute oral bioavailability. Distribution studies have shown that GAL reaches a rapid equilibrium between plasma and tissues, with the highest levels in metabolic organs such as the liver and kidney. Tissue concentrations decreased at a similar rate to plasma, indicating uniform distribution and no undue retention of the compound. Noetzli et al. [43] specified that GAL is predominantly metabolized in the liver by cytochrome P450 (CYP) 2D6 and CYP3A4 enzymes [44].

### 2.5. In Silico Protein Target Prediction of PGEO Biocompounds

**Figure 3:**
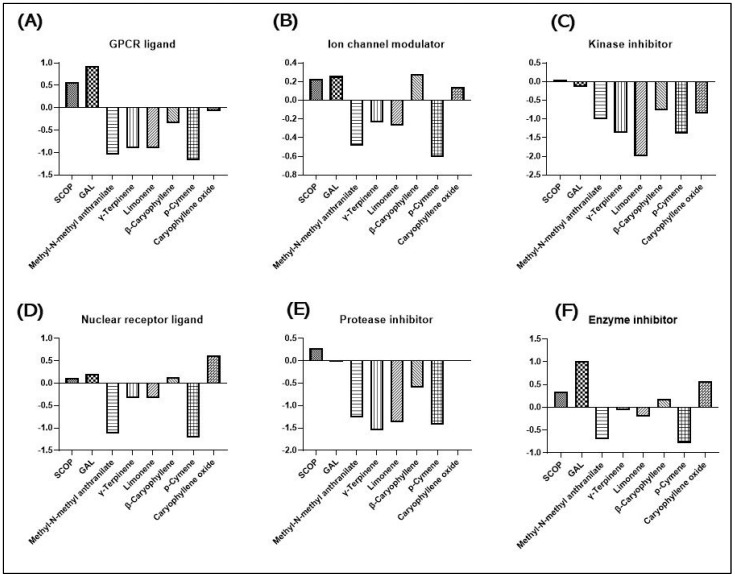
Bioactivity score of SCOP, GAL, Methyl-N-methyl anthranilate, γ-Terpinene, Limonene, β-Caryophyllene, p-Cymene, and Caryophyllene oxide calculated using Molinspiration Cheminformatics software on (**A**) G protein-coupled receptor ligand (GPCR), (**B**) ion channel modulation, (**C**) kinase inhibition, (**D**) nuclear receptor ligand, (**E**) protease inhibitors, and (**F**) enzyme inhibitors.

The determination of bioactivity scores for compounds was performed using Molinspiration cheminformatics software (https://www.molinspiration.com/). In the context of G protein-coupled receptors (GPCRs), the Molinspiration Cheminformatics analysis reveals that SCOP and GAL scored greater than 0.00, indicating their potential to display biologically significant activities on GPCR receptors. On the other hand, methyl-N-methyl anthranilate, γ-terpinene, limonene, β-caryophyllene, β-cymene, and caryophyllene oxide scored less than 0 and are not bound to GPCRs (Figure 3A). In terms of ion channel modulation, SCOP, GAL, β-caryophyllene, and caryophyllene oxide obtained the highest scores (Figure 3B), while methyl-N-methyl anthranilate, γ-terpinene, limonene, and p-cymene obtained scores lower than 0 (Figure 3B). The assay data indicate that none of the compounds have the potential to inhibit the activity of kinases (Figure 3C).

SCOP, GAL, β-caryophyllene, and caryophyllene oxide showed bioactivity scores greater than 0.00 when interacting with the nuclear receptor ligand (Figure 3D). This indicates that these compounds have significant potential for biological activities. On the other hand, methyl-N-methyl anthranilate, γ-terpinenes, limonene, and P-cymene did not show any interaction with the nuclear receptor ligand (Figure 3D). When it comes to protease inhibition, only SCOP and caryophyllene oxide demonstrated significant inhibition potential (Figure 3E). In addition, SCOP, GAL, β-caryophyllene, and caryophyllene oxide showed an ability to inhibit the activity of various enzymes. In contrast, methyl-N-methyl anthranilate, γ-terpinene, limonene, and P-cymene did not possess inhibitory enzyme activity (Figure 3F).

The essential oil extracted from lemon peels has previously been shown to inhibit essential enzymes associated with neurodegenerative disorders (AChE and BChE) and counteract pro-oxidant-induced lipid peroxidation [45]. Kim et al. [46] examined the composition of the essential oil extracted from the peel of Citrus medica L. var. sarcodactylis by using the GC-MS technique and investigated how it acts as an anti-inflammatory agent on mouse macrophage cells exposed to lipopolysaccharide (LPS) (RAW 264.7). The authors revealed 15 different compounds that closely resemble the composition of our oil, with limonene and γ-terpinene being the most abundant. The oil of Citrus medica L. var. sarcodactylis demonstrated a strong ability to inhibit the production of nitric oxide (NO) and prostaglandin E2 (PGE2) by suppressing the synthesis of iNOS and COX-2 proteins, respectively. It also reduced the release of pro-inflammatory cytokines such as TNF-α, IL-1β, and IL-6. Regarding the underlying mechanisms, it was able to inhibit the activation of nuclear factor κB (NF-κB) and JNK and ERK signaling pathways in LPS-treated macrophages. These findings suggest that Citrus oils may have the potential to act as anti-inflammatory agents, providing interesting perspectives for future research in this area.

### 2.6. Evaluation of PGEO Activity on the Anxiety Response

**Figure 4:**
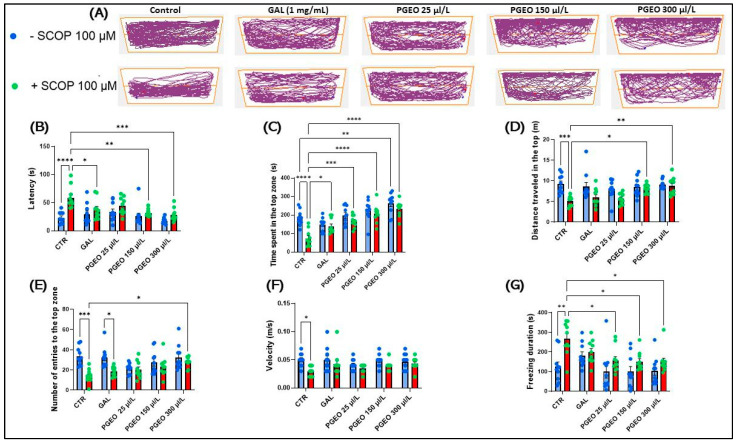
The effects of PGEO (25, 150, and 300 μL/L) administration on locomotion and behavioral parameters in different groups in the NTT. GAL (1 mg/L) was used as a positive reference drug. (**A**) A graphical representation of the swimming pattern in the NTT test; (**B**) latency (s); (**C**) the time spent in the top zone (s); (**D**) the distance traveled in the top zone (m); (**E**) the number of entries to the top zone; (**F**) velocity (m/s); and (**G**) the freezing duration of the zebrafish in the novel tank diving test (NTT). Values are expressed as means ± S.E.M. (n = 10). For Tukey’s post hoc analyses, * *p* < 0.05, ** *p* < 0.01, *** *p* < 0.001, and **** *p* < 0.0001.

Figure 4A shows the differences in locomotor behavior in the novel tank diving test (NTT) between the upper and lower areas of the tank. Groups treated with SCOP (100 µM) preferred the lower area, indicating a high level of anxiety. Tukey’s post hoc analyses showed that acute administration of SCOP treatment for 30 min, occurring immediately before the start of the NTT test, elicited a robust anxiogenic response. This was supported by the significant prolongation of the latency period (*p* < 0.0001) (Figure 4B), the substantial reduction in the time spent by the zebrafish in the top zone of the tank (*p* < 0.0001) (Figure 4C), and the significant increase in the distance traveled by zebrafish in the top zone of the tank (*p* < 0.001) compared to the control group (Figure 4D). Moreover, it was observed that the administration of SCOP (100 μM) induced a hypolocomotor effect in the zebrafish, evidenced by a marked decrease in the number of entries to the top zone of the tank (*p* < 0.001) (Figure 4E), a reduction in velocity (*p* < 0.05) (Figure 4F), and an enhancement of freezing duration (*p* < 0.01) in SCOP-treated zebrafish during the NTT test, which lasted exactly 6 min for each zebrafish tested individually (Figure 4G). These findings imply a decrease in the locomotor activity of the examined animals following administration of SCOP at a concentration of 100 µM. SCOP is a pharmacological agent commonly used as a standard for inducing cognitive deficits in animal studies and even in clinical research involving the assessment of cognitive function in humans. This is an essential reference tool in neuroscience research for the study and understanding of cognitive processes and their associated diseases, such as AD and other memory and learning disorders. Intracerebral injections of SCOP have been shown to affect a wide range of behavioral processes, including taste aversion, anxiety, short-term memory, and attention. This suggests that SCOP influences not only higher cognitive functions but also the emotional and behavioral aspects of brain function [35,47]. In addition, the association of SCOP with a state of anxiety in zebrafish was previously reported in a study [48] indicating that zebrafish treated with SCOP at 120 mg/L and 240 mg/L show fewer entries and greater latency. On the contrary, chronic administration for 8 consecutive days of PGEO, in the analyzed concentrations (25, 150, and 300 μL/L), significantly reduced the latency (*p* < 0.01 for 150 and 300 μL/L) (Figure 4B) and significantly increased the time spent in the upper zone (*p* < 0.001 for 25 and *p* < 0.0001 for 150 and 300 μL/L) (Figure 4C) and the distance traveled in the upper zone of the tank (*p* < 0.001 for 25 and *p* < 0.0001 for 150 and 300 μL/L) (Figure 4D). Therefore, our results indicate the potential of PGEO to ameliorate anxiety in amnesic zebrafish. In addition to these analyses, our results also indicate that PGEO can have beneficial effects on the general locomotor activity of zebrafish with hypolocomotor activity due to the administration of SCOP (100 µM) in water. Thus, in the fish from the group treated with PGEO 300 μL/L and subsequently subjected to acute SCOP treatment, an increase in the number of entries to the top zone (*p* < 0.05) (Figure 4E) was observed, as well as a reduction in total freezing duration in all three concentrations analyzed (*p* < 0.05) (Figure 4G); however, PGEO was found to be ineffective in restoring velocity (Figure 4F).

Tukey’s post hoc analyses did not identify significant differences in zebrafish chronically treated with PGEO (25, 150, and 300 μL/L) (they did not undergo acute SCOP treatment) compared to the control group or groups of zebrafish that had received PGEO in similar concentrations plus SCOP (100 µM), except for treatment with PGEO in the concentration of 300 µL/L which managed to intensify the exploration time in the top zone compared to the control group (*p* < 0.01) (Figure 4G). The essential oil derived from the peel of *Citrus aurantium* L. is frequently used as a popular alternative for treating anxiety and other CNS disorders. The main compounds in this oil, limonene, and myrcene, are recognized for their biological activity on the CNS. Previous studies have demonstrated the anxiolytic activity of this oil, manifested by increasing the time mice spent in the open arms of a plus maze [49] or within a light/dark maze [50]. It is assumed that this biological effect can be attributed either to a specific compound in the composition or, more likely, to a synergistic effect between several compounds present in the essential oil. The results of a meta-analysis revealed that *Citrus* essential oils provide neuroprotective and anti-aging effects, relieving respiratory congestion, pain, insomnia, anxiety, depression, stress, and other psychological and physiological disorders, mainly due to their antioxidant properties [51].

### 2.7. Evaluation of PGEO Impact on Spatial Memory and Response to Novelty

**Figure 5:**
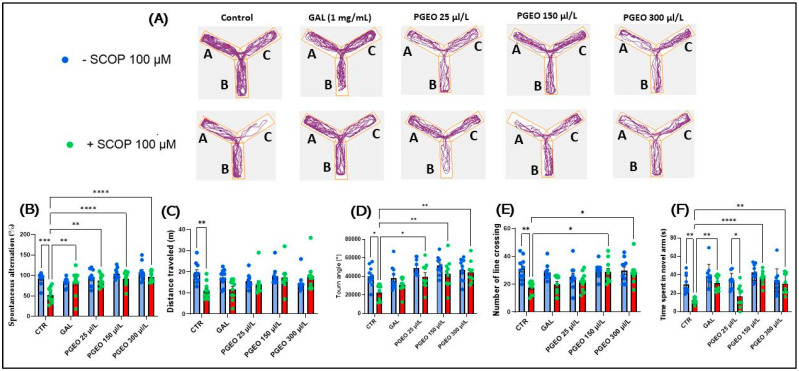
The effects of PGEO (25, 150, and 300 μL/L) administration on spatial memory and responses to novelty in the Y-maze test. GAL (1 mg/L) was used as a positive reference drug. (**A**) A graphical representation of zebrafish swimming behaviors in the Y-maze; (**B**) spontaneous alternation (%); (**C**) distance traveled (m); (**D**) turn angle (°); (**E**) number of line crossing; and (**F**) time spent in the novel arm (s). Values are expressed as means ± S.E.M., (n = 10). For Tukey’s post hoc analyses, * *p* < 0.05, ** *p* < 0.01, *** *p* < 0.001, and **** *p* < 0.0001.

Figure 5A shows the swimming patterns in the Y-maze test and reveals memory differences between the groups. The zebrafish treated with SCOP (100 μM) spent more time in the familiar arm and less time in the novel arm, suggesting that they have difficulty remembering which arm they previously explored.

Post hoc analyses using Tukey’s method demonstrated that acute administration of SCOP treatment, performed for 30 min before the Y-maze test, resulted in both impairment of general locomotor activity and disruption of orientation in the zebrafish. This was reflected by a decrease in the percentage of spontaneous alternation (%) (*p* < 0.001) (Figure 5B), distance traveled (*p* < 0.0001) (Figure 5C), turn angle (°) (*p* < 0.05) (Figure 5E), limited number of line crossing (*p* < 0.01) (Figure 5E), and reduced time spent in the novel arm (*p* < 0.01) (Figure 5F) compared to the control group. SCOP has previously been shown to influence recordings of spatial information captured by miniscope calcium imaging in mouse hippocampal cells. Studies in animal models such as mice and rats have shown that SCOP administration can lead to significant deficits in memory consolidation and is associated with impairment of spatial learning and memory in maze tests used to assess cognitive functions in animals [52,53,54]. In addition, SCOP has been reported to cause learning and memory impairment in zebrafish [55]. A return to the normal movement patterns of the zebrafish in the Y-maze test was observed in the groups of zebrafish which, apart from the acute exposure for 30 min to SCOP, were also exposed to chronic treatment with PGEO (25, 150, and 300 μL/L). Thus, the chronic exposure of zebrafish for 11 days to PGEO changed their movement patterns, improving their locomotor activity by increasing the turning angle (°) (*p* < 0.05 for 25 and *p* < 0.01 for 150 and 300 μL/L) (Figure 5D) and the amplification of the number of line crossing (*p* < 0.05 for 150 and 300 μL/L) (Figure 5E). Simultaneously, PGEO also showed positive effects on spatial memory in the zebrafish from SCOP-treated groups by restoring the percentage of spontaneous alternation (*p* < 0.001 for 25 and *p* < 0.01 for 150 and 300 μL/L) (Figure 5D) and time spent by the zebrafish in the novel arm of the Y-maze test (*p* < 0.01 for 300 and *p* < 0.0001 for 150 μL/L) (Figure 5F). However, Tukey’s post hoc analyses revealed no significant difference between fish exposed to chronic PGEO treatment alone (25, 150, and 300 μL/L) compared to the control group or with groups of zebrafish receiving PGEO in comparable concentrations alongside SCOP (100 μM). GAL (1 mg/L) was used as a positive reference drug. Tukey’s post hoc analyses demonstrated that GAL induced improvements in spatial memory in SCOP-treated fish by increasing spontaneous alternation (*p* < 0.01) (Figure 5B) and the time spent in the novel arm (*p* < 0.01) (Figure 5F). Ueda et al. [56] suggested that inhalation of lemon essential oil has a significant impact on working memory task performance and brain activity recorded by electroencephalography, which showed activation of delta and theta bands in various regions of the prefrontal cortex, including the anterior cingulate gyrus and the orbitofrontal, as well as in other regions such as the superior temporal gyrus, the parahippocampal gyrus, and the insula. Persistent alpha band activation was also observed in the prefrontal cortex, including the anterior cingulate gyrus. Furthermore, Liu et al. [57] showed that lemon essential oil improves spatial memory in APP/PS1 mice as well as wild-type C57BL/6L mice by reducing AChE levels and increasing BDNF, PSD95/synaptophysin levels, and by improving synaptic plasticity.

### 2.8. Evaluation of the PGEO Impact on Recognition Memory

**Figure 6:**
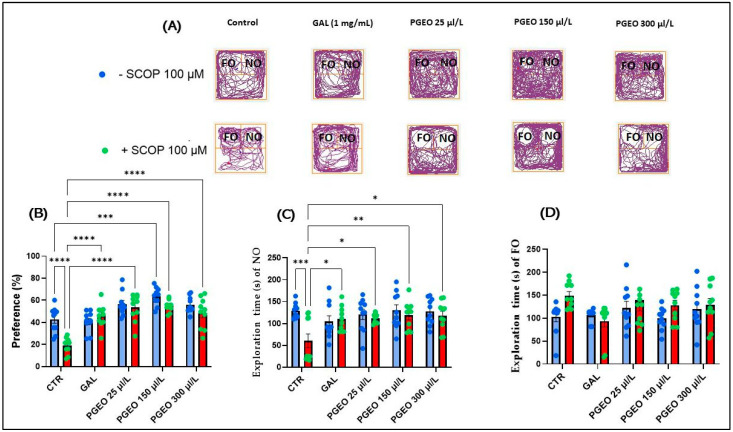
The effects of PGEO (25, 150, and 300 μL/L) administration on recognition memory in the NOR test. GAL (1 mg/L) was used as a positive reference drug. (**A**) A graphical representation of zebrafish track plots during the test session from the NOR test. The familiar object area is denoted by the initials (FO) and the novel object area is denoted by the initials (NO); (**B**) percent preference (%) to one of the two objects in the NOR test; (**C**) exploration time (s) of the NO; and (**D**) exploration time (s) of the FO. Values are expressed as means ± S.E.M., (n = 10). For Tukey’s post hoc analyses, * *p* < 0.05, ** *p* < 0.01, *** *p* < 0.001, and **** *p* < 0.0001.

Figure 6A illustrates the differences in familiar object (FO) and novel object (NO) exploration. In the novel object recognition test (NOR), the typical locomotion pattern indicates that the SCOP-treated group did not show a preference for the two objects tested, indicating the induction of memory deficits following acute exposure of the fish to SCOP (100 μM).

Tukey’s post hoc analyses indicated a significant decrease in preference percentage in the zebrafish exposed for 30 min with SCOP (100 µM) before testing compared to the control group (*p* < 0.0001) (Figure 6B), and an impairment of ON exploration time (*p* < 0.001) (Figure 6C), indicating that SCOP has an amnesic effect on recognition memory in zebrafish. In contrast, chronic administration for 18 days of PGEO to zebrafish that were subsequently subjected to acute treatment with SCOP significantly increased the percentage preference (%) at all concentrations analyzed (*p* < 0.001) in a similar manner to that of GAL (*p* < 0.0001) (Figure 6B). Simultaneously, in the fish from the groups treated with PGEO (25, 150, and 300 μL/L) + SCOP (100 μM), an increase in NO exploration time was also observed (*p* < 0.05, for 25 and 300 and *p* < 0.01 for 150 μL/L) (Figure 6C). In addition, Tukey’s post hoc analyses also did not reveal a significant difference in preference (%) to the NO of native fish that were only exposed to chronic PGEO treatment at the concentration of 300 μL/L and the fish in the group control (*p* < 0.01) (Figure 6B). Therefore, our data show for the first time that PGEO could have beneficial effects on zebrafish’s recognition memory and also restore memory in animals exposed to anticholinergic agents. Braidy et al. [58] described nobiletin and tangerine as two flavonoids isolated from Citrus peel with potential neuroprotective effects, which alleviate cholinergic deficits, reduce the accumulation of amyloid-beta peptides, counteract N-methyl-D-aspartate (NMDA) receptor hypofunction, improve ischemic lesions, inhibit tau protein hyperphosphorylation, increase neprilysin levels, modulate various signaling cascades, and protect against toxicity induced by substances such as 1-methyl-4-phenylpyridinium (MPP(+)) and 1-methyl-4-phenyl-1, 2,3,6-tetrahydropyridine (MPTP).

### 2.9. Effects of PGEO on AChE Activity

**Figure 7:**
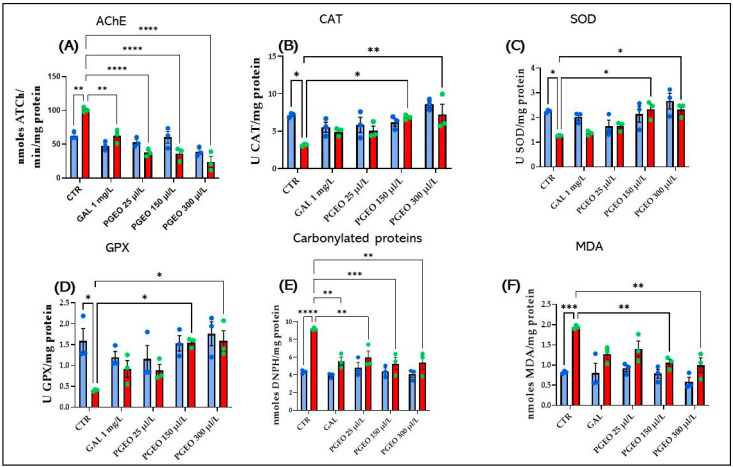
The effects of PGEO (25, 150, and 300 μL/L) administration on (**A**) acetylcholinesterase (AChE); (**B**) superoxide dismutase (SOD); (**C**) catalase (CAT); and (**D**) glutathione peroxidase (GPX) specific activities; (**E**) carbonylated protein levels and (**F**) the malondialdehyde (MDA) content. GAL, (1 mg/L) was used as a positive control. Values represent means ± SEM (n = 3) followed by Tukey’s post hoc analyses: * *p* < 0.05, ** *p* < 0.01, *** *p* < 0.001, and **** *p* < 0.0001.

Figure 7A illustrates that acute treatment with SCOP (100 μM) significantly enhanced AChE activity in zebrafish’s brains compared to the control group (*p* < 0.01). The present data follow the studies of Sun et al. [59], Baek [60], and Kim [61] which reported that SCOP produces significant cholinergic deficits and increases AChE activity in the hippocampus, thus contributing to cerebral neurodegeneration.

Spectacularly, the exposure of zebrafish to chronic treatment with PGEO (25, 150, and 300 μL/L), before exposure to SCOP for 30 min significantly reduced AChE activity in all the concentrations established within the analysis (*p* < 0.0001), in a similar way to GAL (*p* < 0.001). This suggests that PGEO could increase ACh levels in zebrafish’s brains subjected to anticholinergic agents, potentially enhancing cholinergic neurotransmitter function and indicating its potential regulator in cholinergic neurotransmission. Surprisingly, in zebrafish from the groups that received chronic treatment only with PGEO (25, 150, 300 µL/L) before the acute treatment with SCOP (100 µM), there were no changes in brain AChE activity compared to the groups of zebrafish treated with both PGEO and SCOP or the control group. Therefore, PGEO could prevent the degradation of cholinergic neurotransmitters, leading to decreased AChE levels in the brains of amnesic zebrafish, and regulate ACh levels, which are depleted following SCOP administration.

Previous studies have similarly indicated that several essential oils extracted from different Citrus fruits exhibit strong anti-AChE activity [11,62,63]. Simultaneous, Oyeniran et al. [64] demonstrated that alkaloid-rich extracts from lemon leaves (Citrus limon) can have beneficial effects on cognitive and memory functions assessed by Morris water maze, Y-maze, and open field maze tests in laboratory animals, especially in rats with SCOP-induced amnesia, by regulating AChE activity. Aumeeruddy-Elalfi et al. [65] showed that essential oils extracted from six medicinal and food plants exhibited the ability to inhibit enzymes involved in various conditions, including diabetes, skin aging, and neurodegenerative disorders. Among these enzymes, AChE was strongly inhibited by essential oils from Citrus hystrix and Citrus reticulata, and the inhibition was more effective than that obtained with GAL. Essential oils have also demonstrated the ability to inhibit other enzymes, such as collagenase, elastase, and alpha-glucosidase.

### 2.10. Effects of PGEO on Brain Oxidative Stress

An antioxidant defense system based mainly on the enzymes CAT, SOD, GPX, and GSR protects the body and its tissues from cellular damage induced by oxidative stress [66]. Multiple studies have repeatedly demonstrated that oxidative stress contributes to the etiology of multiple neurodegenerative disorders, including AD [67,68]. According to Tukey’s post hoc analyses, compared to the control group, SCOP caused a significant decrease in the activity of CAT (*p* < 0.05) (Figure 7B), SOD (*p* < 0.05) (Figure 7C), and GPX (*p* < 0.05) (Figure 7D), as well as an increase in protein carbonyl content (*p* < 0.0001) (Figure 7E) and MDA levels in zebrafish’s brains (*p* < 0.001) (Figure 7F). Conversely, chronic treatment with PGEO (25, 150, and 300 μL/L) showed antioxidant effects in zebrafish’s brains, being able to restore the level of enzymes with antioxidant activity and decrease the oxidative pathogenicity induced by SCOP. These protective effects of PGEO on oxidative stress are due to its ability to enhance the specific activity of CAT (*p* < 0.05 for 150 μL/L and *p* < 0.001 300 μL/L) (Figure 7B), SOD (*p* < 0.05 for 150 and 300 μL/L) (Figure 7C), and GPX (*p* < 0.05, 150 μL/L and 300 μL/L) (Figure 7D). Also, daily chronic administration of PGEO proved to be effective against protein carbonylation (*p* < 0.01 for 25 μL/L and 300 μL/L and *p* < 0.001 for 150 μL/L), having an effect similar to that shown by the acute administration of GAL (*p* < 0.01) (Figure 7E). Simultaneously, a decrease in MDA content (*p* < 0.01 for 150 and 300 μL/L) was also observed in zebrafish exposed to chronic treatment with PGEO and then to acute treatment with SCOP (Figure 7F) compared to zebrafish that received only acute treatment with SCOP. However, no statistically significant differences were revealed between the zebrafish treated only with PGEO and those treated chronically with PGEO and then exposed to acute administration of SCOP treatment for 30 min. Therefore, PGEO attenuates SCOP-induced oxidative stress, demonstrating a protective effect, and has no adverse effects on oxidative status in native zebrafish. The antioxidant effects of Citrus essential oils on the brains of laboratory animals have been previously reported in multiple studies [69,70,71,72,73].

A variety of oxygen free radicals can be seen in the human brain, including the amino superoxide radical (O_2−_), hydrogen peroxide (H_2_O_2_), hydroxyl radical (OH), and nitric oxide (NO). Although O_2−_ is not considered to be one of the most reactive oxygen species (ROS), it possesses the ability to oxidize transition metal ions, thus causing the deactivation of antioxidant enzymes that rely on these metals as cofactors. Moreover, it can oxidize cysteine, resulting in changes in the protein structure and a consequent reduction in the bioactivity of certain enzymes. A considerably more powerful antioxidant is the protonated version of the superoxide anion radical, (HO_2−_). This radical can penetrate cell membranes and serves as the primary initiator of protein oxidation and lipid peroxidation [74,75]. In the case of the establishment of oxidative stress, the primary cellular impact comes from the damage caused to macromolecules due to the presence of free radicals. These macromolecules include polyunsaturated fatty acids found in lipid membranes, crucial proteins, and DNA. Lipid peroxidation, which is the process by which tissue damage is induced by free radicals, has been linked to several pathological conditions, including brain disorders. This peroxidation is a significant consequence of free radical damage, directly affecting cell membranes and producing various by-products such as aldehydes (MDA) and ketones. Free radicals exert their cytotoxic effect by peroxidizing the phospholipids in cell membranes, thus changing their permeability, increasing their fluidity and stiffness, and, in certain cases, causing loss of integrity, which increases the risk of membrane rupture. MDA, being the most abundant resulting aldehyde, is considered a marker for this process, and an increase in its content signifies an increase in oxidative stress [76,77]. Cells develop an enzymatic antioxidant pathway to combat oxidative stress. The initial step of this pathway involves the catalytic conversion of superoxide radicals to hydrogen peroxide by SOD. Immediately, CAT and GPX are activated to remove hydrogen peroxide. It is important that antioxidant enzymes effectively neutralize any excess hydrogen peroxide resulting from an increase in the catalytic activity of SOD, which may be achieved by CAT or GPX [75,76]. Furthermore, the occurrence of neuronal death is a consequence of the deleterious impact of oxidative stress on lipids, proteins, and DNA. This process is closely related to AD. Moreover, apoptosis can be initiated by oxidative stress by modulating the ERK1/2 and Nrf2 signaling pathways. This is followed by an increase in GSK-3β expression and a decrease in PP2A activity. Disease progression is exacerbated by oxidative stress as it interferes with various signaling pathways such as RCAN1, CREB/ERK, Nrf2, PP2A, NFκB, and PI3K/Akt [78].

In a photochemical study, methyl-N-methyl anthranilate was characterized as having antioxidant properties and being able to act on reactive oxygen species [79]. γ-Terpinene has also been associated with robust antioxidant effects, being able to inhibit lipid peroxidation [80]. Data from another study revealed that Shiikuwasha essential oil, rich in limonene and γ-terpinene, alleviates physiological stress and exhibits anti-inflammatory activity. Administration of it and its components reduced work errors and induced positive effects on heart rate variability and brain activity in women suffering from physiological stress [81]. β-Caryophyllene was investigated by Ojha et al. [82] for its neuroprotective effects in a rat model of Parkinson’s disease (PD) induced by rotenone. β-Caryophyllene reduces oxidative stress and inflammation, saving dopaminergic neurons and inhibiting the activation of microglia and astrocytes. Also, p-cymene has significant antioxidant effects on protein and lipid oxidation [83].

### 2.11. Correlation Analyses between Behavioral and Biochemical Parameters

Pearson’s correlation coefficient (r) was used to explore the relationships between behavioral scores, enzyme activities, and lipid peroxidation, which included variables such as latency required for the zebrafish to start vertical swimming up the water column towards the upper zone of the tank in the NTT test, time spent in the novel arm of the Y-maze test, preference (%) in the NOR test, AChE, SOD, CAT, and GPX specific activities, and level carbonylated protein levels in zebrafish’s brains relative to MDA level.

**Figure 8:**
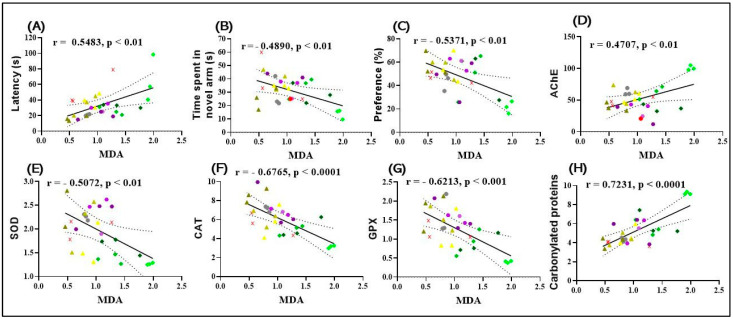
Correlation analysis between behavioral and biochemical parameters (Pearson’s correlation). Data shown are (**A**) latency to entry in top zone of the NTT and MDA test (n = 3, r = 0.5483, *p* < 0.01); (**B**) time spent in the novel arm of the Y-maze and MDA (n = 3, r = −0.4890, *p* < 0.01); (**C**) preference (%) in the NOR test over MDA (n = 3, r = −0.5371, *p* < 0.01); (**D**) AChE vs. MDA (n = 3, r = 0.4707, *p* < 0.001); (**E**) SOD vs. MDA (n = 3, r = −0.5702, *p* < 0.01); (**F**) CAT vs. MDA (n = 3, −0.6765, *p* < 0.0001); (**G**) GPX vs. MDA (n = 3, r = −0.6213, *p* < 0.001); and (**H**) carbonylated proteins vs. MDA (n = 3, r = 0.7231, *p* < 0.001), (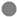) control, (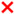) Galantamine (GAL, 1 mg/mL), (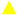) petitgrain essential oil (PGEO, 1 μL/L), (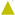) PGEO 3 μL/L, (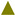) PGEO 6 μL/L, (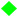) Scopolamine (SCOP, 100 μM), (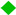) SCOP (100 μM) + GAL 1 mg/mL, (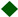) SCOP (100 μM) + PGEO 1 μL/L, (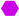) SCOP (100 μM) + PGEO 3 μL/L, and (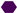) SCOP (100 μM) + PGEO 6 μL/L.

The results showed a significant positive correlation between the time spent in the upper zone in the NTT and MDA (r = 0.5483; *p* < 0.01) (Figure 8A). Also, time spent exploring the novel arm of the Y-maze (r = −0.4890; *p* < 0.01) (Figure 8B) and preference (%) in the NOR test (r = −0.5371; *p* < 0.01) (Figure C) were negatively associated with MDA content. The specific activity of CAT (r = −0.6765; *p* < 0.0001) (Figure 8F), the specific activity of SOD (r = −0.5072; *p* < 0.01) (Figure 8E), and the specific activity of GPX (r = −0.4890; *p* < 0.01) (Figure 8G) were reported with the level of MDA.

At the same time, there was a significant positive correlation between the specific activity of AChE (Figure 8D) and the level of carbonylated proteins (Figure 8H) with the level of MDA, with correlation coefficients r between 0.4707 and 0.7231. These results suggest a complex interaction between behavior, enzyme activities, and lipid peroxidation, bringing to light the pathophysiological mechanisms involved in treatment response and their effects on the nervous system.

Given its impact on markers of oxidative stress and improvements seen in behavioral tests, PGEO could represent a promising option for treating symptoms associated with amnesia and anxiety.

## 3. Materials and Methods

### 3.1. Plant Material and Essential Oil Preparation

PGEO was prepared by water distillation on the leaves of *Citrus reticulata* using the Clevenger apparatus for 5 h. Its color was pale yellow and had an intensely sweet and fresh scent. The oil was purchased from Somitt Aromatic Company and was kept in dark bottles.

### 3.2. Gas Chromatography/Mass Spectrometry (GC/MS) Analysis

The chromatographic analysis of PGEO was conducted with a Hewlett Packard gas chromatograph (GC HP 5890 II; Hewlett Packard GmbH, Bad Homburg, Germany). A Rtx-5MS fused-bonded silica column (30 m × 0.25 mm i.d.; film thickness 0.25 mm; Ohio Valley, OH, USA) was used. The capillary column was directly coupled to a quadrupole mass spectrometer (SSQ 7000; Thermo-Finnigan, Bremen, Germany). The injector temperature was 250 °C. The Helium carrier gas flow rate was 2 mL/min. All the mass spectra were recorded with the following analytical conditions: filament emission current, 60 mA; electron energy, 70 eV; ion source temperature, 200 °C; and a scan range from 40 to 400 Amu. The diluted samples (0.5% *v*/*v* n-hexane used as solvent) were injected through the split mode (split ratio, 1:15). Compounds were identified by comparison of their mass spectral data and retention indices were identified through the Wiley Registry of Mass Spectral Data 8th edition and the NIST Mass Spectral Library (December 2005). The identification was further confirmed by the calculation of the retention indices (RIs) relative to a homologous series of n-alkanes (C6–C22), under identical experimental conditions, as well as matching with the literature [84].

### 3.3. Biomedical Alert and Structural Prediction of Biocompounds from PGEO, Drug Analogy

The canonical system—the Simplified Molecular Input Line Entry System (SMILES)—is a simplified method of representing chemical structures, used in computational analysis and other fields related to chemistry and biochemistry. The SMILES is a way to describe chemical structures in a text format that can be easily interpreted by computers and other software programs. The main advantages of using the SMILES include ease of use, compactness, and the ability to be automatically interpreted by software programs. This makes the SMILES useful in a variety of applications, including computational analysis of chemical compounds, drug design, molecular modeling, and other fields [85,86].

In the computational analysis, we opted to use the canonical SMILES to simplify the representation of chemical structures and examine the main biocompounds identified in PGEO, such as methyl-N-methyl anthranilate (89.93%), γ-terpinene (6.25%), limonene (1.35%), β-caryophyllene (1.11%), p-cymene (0.72%), and caryophyllene oxide (0.63%), compared to SCOP and GAL (Table 5).

To assess the structural parameters essential for effective action within the body of the analyzed compounds, we employed the SwissADME online tool (http://www.swissadme.ch/index.php, accessed on 13 October 2023). This platform facilitated the computation of key principles in drug design, including Lipinski’s Rule [24], Weber’s Rule [87], Ghose’s Rule [26], and Egan’s Rule [27]. These computational methodologies rely on algorithms trained on extensive datasets and demonstrate considerable accuracy following rigorous cross-validation. Lipinski’s Rule encompasses the following criteria: maintaining a molecular mass under 500 Daltons, limiting the maximum number of hydrogen bond donors to 10, capping the maximum number of hydrogen bond acceptors at 5, and ensuring that the octanol/water logarithm (Log P(o/w)) remains below 5. Weber’s Rule evaluates the number of rotational bonds, also known as molecular complexity, within a molecule. Ghose’s Rule outlines the following criteria: a molecular mass ranging between 160 and 480 Da, an octanol/water logarithm (Log P(o/w)) falling within the range of 0.4 to 5.6, a refractive index between 40 and 130, and a total atom count ranging from 40 to 130, serving as a molecular descriptor. Egan’s Rule primarily focuses on liver toxicity and relies on parameters such as the Liver Toxicity Index, calculated based on the specific chemical properties of the molecule, which must fall below a specified threshold. Additionally, it considers the ratio of water solubility to octanol solubility (Log P(o/w)), which should fall within a defined range to indicate a reduced likelihood of liver toxicity.

### 3.4. In Silico Estimation of Pharmacokinetic Profile of Biocompounds from PGEO

For the computational analysis, the SMILES was used for SCOP, GAL, and the main compounds of PGEO (methyl-N-methyl anthranilate, γ-terpinene, limonene, β-caryophyllene, p-cymene, and caryophyllene oxide), which were retrieved from the PubChem platform [88], in January 2024. Two free calculators (SwissADME (http://www.swissadme.ch/index.php, accessed on 13 October 2023) [89] and pKCSM (https://biosig.lab.uq.edu.au/pkcsm/, accessed on 13 October 2023) [90]) were used. To make a comparison between the predictions obtained using the two free online platforms and the experimental data, it was imperative to standardize the units of measurement, such as solubility, log S, and clearance. Regarding other parameters, such as absorption and permeability, it was necessary to convert the data into binary categories (e.g., Yes/No). This standardization was essential to facilitate the evaluation of predictions, as some sites used different levels of classification. Through this approach, we ensured the consistency and comparability of the data, providing a uniform framework for analyzing and interpreting the results.

We selected the following probabilities for analysis: 1. intestinal absorption (human), 2. skin permeability, 3. steady-state volume of distribution (VDss) (human), 4. unbound fraction (human), 5. blood–brain barrier permeability (BBB), 6. central nervous system (CNS) permeability, 7. substrate for CYP3A4, 8. inhibitor for CYP1A2, 9. total clearance, 10. substrate for the renal organic cation transporter (OCT2), 11. maximum dose tolerated (human), and 12. hepatotoxicity. These probabilities were selected to evaluate various aspects related to the behavior and interactions of the compounds in the human body, thus providing essential information for evaluating their therapeutic potential and the safety of their use in medicine.

### 3.5. In Silico Protein Target Prediction for PGEO Compounds

To increase understanding and obtain a more accurate prediction of the protein targets of the investigated compounds, the Molinspiration platform was used. The use of Molinspiration (https://www.molinspiration.com/, accessed on 13 October 2023) was crucial in the calculation of the medicinal activities of the analyzed compounds. The bioactivity score has been an essential metric used to evaluate the potency of molecules in terms of their biological activity. A score exceeding 0.00 indicates substantial biological activity, while scores between −0.50 and 0.00 imply moderate activity. Scores below −0.50 indicate a reduced likelihood of biological activity. These assessments provided a deeper understanding of the compounds’ therapeutic potential, helping to identify the most promising candidates for further screening and drug development [91].

### 3.6. Experimental Animals

This study explored the potential neuroprotective effects of PGEO on zebrafish. Specifically, we aimed to assess its impact on cognitive function, anxiety-like behavior, and oxidative stress markers in these animals. We employed 100 adult Tubingen wild-type zebrafish (Danio rerio), aged between 4 and 6 months. To ensure genetic consistency and eliminate potential confounding variables related to fin morphology, wild-type fish were chosen. A balanced sex ratio (1:1 male to female) was maintained within the study population. The average body length of the zebrafish ranged from 3 to 4 cm.

### 3.7. Animal Care and Housing

To prioritize animal welfare, all zebrafish were purchased from the European Zebrafish Resource Center at the Institute of Toxicology and Genetics, Germany. Upon arrival, they underwent a two-week quarantine period in a 60 L tank to monitor their health and acclimatize them to the laboratory environment. Following quarantine, the zebrafish were housed in groups of 10 individuals in a transparent glass tank with a capacity of 10 L each. To ensure optimal water quality, dechlorinated water treated with Tetra AquaSafe (Tetra, Germany) was used. Regular water changes were performed every two days to maintain a clean and healthy environment for the fish. The water temperature was meticulously maintained at 28 °C ± 2 °C, mimicking a tropical environment suitable for zebrafish. Water quality parameters were rigorously monitored twice daily (8 a.m. and 6 p.m.) to ensure optimal conditions for the fish. These parameters included the following: pH: 7.0–7.5 (providing a slightly neutral to slightly alkaline environment), dissolved oxygen: 8 mg/L ± 1 mg/L (critical for maintaining healthy oxygen levels for fish respiration), conductivity: 1500–1600 µS/cm (indicating a suitable range of dissolved ions for proper physiological function), and ammonia/nitrite levels: below 0.001 mg/L (ensuring minimal presence of toxic nitrogenous waste products). A standard lighting cycle of 14 h light and 10 h dark was implemented to replicate a natural day/night pattern. To maintain proper nutrition and health, the zebrafish were fed Norwin Norvital flakes (Norwin, Denmark) three times a day (at 8 a.m., 1 p.m., and 8 p.m.). The feeding amount was carefully controlled, ensuring the fish could consume all the food within 10 min to minimize waste and maintain water quality. Notably, the air pump was turned off during feeding times to facilitate easy food consumption by the fish.

### 3.8. Ethical Considerations

All procedures involving the zebrafish strictly adhered to the guidelines set forth by European Parliament Directives 2010/63/EU. Furthermore, the research protocol received prior approval from the Animal Research Ethics Committee at the Faculty of Biology, Alexandru Ioan Cuza University of Iasi, Romania (approval number: 1714/06.07.2023). This commitment to ethical animal research practices underlines our dedication to responsible scientific investigation.

### 3.9. Experimental Animals and Method of Work

**Figure 9:**
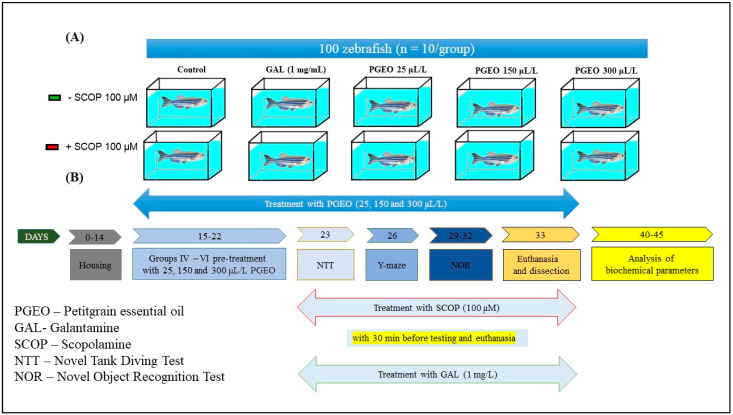
A schematic representation of the experimental design of the study. Section (**A**) illustrates the various experimental groups employed in the research, while (**B**) delineates the behavioral and biochemical tests conducted.

This study employed a randomized controlled trial design with 10 experimental groups of zebrafish (Figure 9A). Each group received a specific treatment as follows:

Group I (control): no treatment.

Group II (positive control): Galantamine (GAL, 1 mg/L) for 3 min before behavioral tests and euthanasia. GAL is a known AD treatment and served as a benchmark for comparison.

Groups III–V (PGEO treatment): chronic exposure to different concentrations of PGEO (25, 150, and 300 μL/L) dissolved in 1% Tween 80 solution, administered through water changes.

Group VI (Scopolamine model): Scopolamine (SCOP, 100 μM) treatment for 30 min before specific behavioral tests and euthanasia. SCO is known to impair memory and induce an amnesic state, mimicking dementia symptoms.

Group VII (SCOP + GAL): SCOP (100 μM) followed by GAL (1 mg/L) 30 min later, similar to Group II (positive control) but with prior SCOP exposure.

Groups VIII-X (SCOP + PGEO): Scopolamine (100 μM) followed by chronic exposure to different PGEO concentrations (25, 150, and 300 μL/L) like Groups III–V.

### 3.10. Animal Treatment

Treatment doses were chosen based on previous research [92,93]. SCOP was administered 30 min before the NTT and euthanasia procedure in Groups VI, VII, and VIII–X. Also, SCO was administered 30 min after the training session in Groups VI, VII, and VIII–X for the Y-maze and NOR tests. Group VII received additional GAL treatment after SCOP to assess its ability to counteract amnesic effects. PGEO (25, 150, and 300 μL/L) was administered chronically, once a day, during the water change. In addition, groups (I, II, VI, and VII) also received chronic treatment with 100 μL of 1% Tween 80 solution, daily, along with the water change (Figure 9).

### 3.11. Behavioral Analysis

In our experimental setup, we employed advanced technology to assess the effects of PGEO on zebrafish behavior. To accomplish this, we utilized a Logitech HD Webcam C922 Pro Stream digital camera, renowned for its high-resolution imaging capabilities, with a full HD resolution of 1080 pixels and a smooth frame rate of 30 per second. This camera, manufactured by Logitech, Lausanne, Switzerland, provided us with detailed video recordings of the zebrafish’s activity. Following the recording process, we conducted a comprehensive analysis of the acquired videos using the ANY maze^®^ software, version 6.3, provided by Stoelting Co., Wood Dale, IL, USA. This software is specifically designed for behavioral analysis in laboratory settings, offering sophisticated tools for data interpretation and visualization.

#### 3.11.1. An Assessment of Anxiety State Using the Novel Tank Diving Test (NTT)

Zebrafish naturally exhibit anxiety when introduced to unfamiliar environments, making them suitable subjects for anxiety-related studies. Various tests have been developed to assess these behaviors, with NTT being particularly well suited. The NTT capitalizes on the zebrafish’s instinctual diving response and has solid face validity, meaning it effectively measures anxiety levels [94,95,96]. In this study, we aimed to evaluate the impact of PGEO on anxiety levels in the acute treatment of SCOP (100 µM)-induced zebrafish model in the NTT. The protocol established by Cachat [97] was followed. For this, we utilized a tank with trapezoidal glass walls, ensuring perfect transparency. The dimensions of the tank were as follows: a height of 15.1 cm, a base length of 23.9 cm, a top length of 28.9 cm, and a width of 6.1 cm. The tank was divided horizontally into two equal sections: a top zone and a bottom zone.

During the test, each zebrafish specimen was individually assessed once for a duration of 360 s. Within this timeframe, we measured anxiety-related behaviors, including latency period (s), time spent in the top area of the tank (s), and distance traveled in that region (m). Additionally, locomotion-related phenotypes of zebrafish were evaluated by the number of entries to the top zone, the recorded freezing duration (s), and the mean swimming speed (velocity) (m/s). These comprehensive measurements allowed us to investigate both the anxiety behaviors and locomotor responses of the fish in the new environment.

#### 3.11.2. An Assessment of Spatial Memory and Response to Novelty Using the Y-Maze

The zebrafish model is an invaluable pharmacological model for investigating the performance of learning and memory, encompassing a range of behavioral parameters. Although the zebrafish model provides insights into memory mechanisms, it primarily facilitates the examination of three common types of memory: spatial, recognition, and associative memory [98]. Typically, memory assessment involves a delay period between the training and testing phases to effectively evaluate memory retention [99].

The Y-maze test presents numerous benefits for the evaluation of memory. It offers a direct and effective approach for assessing memory, permitting expedited training sessions without intricate conditioned learning. Additionally, the Y-maze test reduces the influence of potential confounding variables such as emotional and motivational states, thus enabling a concentrated assessment of memory and behavioral reactions within a regulated setting [98].

To explore the impact of PGEO on spatial memory and the response to novelty in zebrafish, we implemented the Y-maze test according to the established protocol by Cognato et al. [100]. The Y-maze was constructed with transparent glass in the shape of the letter Y, consisting of three arms of equal dimension. The floor of the aquarium was covered with white plastic material, while the other areas were adorned with black plastic, featuring visual cues such as squares, circles, and triangles.

During the training session, the fish were allowed to investigate two arms (start and familiar) for 5 min, while the third arm (novel) remained inaccessible. The central area of the maze, referred to as the preselection zone, was not included in the analysis of the results. Subsequently, the test session took place one hour after the training session, during which the fish were placed in the start arm with access to all three arms for 5 min. The response to novelty was evaluated by observing and measuring the spontaneous alternation (%), turn angle (°), and time spent in the novel arm (s). We also evaluated the locomotor activity by recording the total distance traveled (m) and number of line crossings using a camera positioned above the tank.

#### 3.11.3. The Novel Object Recognition Test (NOR): Assessing Reinstatement Memory in Zebrafish

The NOR test plays a crucial role in studying memory processes in various organisms, including zebrafish. This test offers valuable insights into (a) memory acquisition (formation of new memories), (b) memory consolidation (strengthening and stabilization of memories over time), (c) memory reconsolidation (reinstatement of weakened memories through retrieval) and (d) re-acquisition (learning information previously encountered but forgotten) [101].

To investigate whether PGEO affects zebrafish’s reinstatement memory, we employed the NOR test, following the protocol established by Stefanello et al. [102]. For this experiment, a glass tank in the shape of a cube was utilized, with a width and height measuring 0.3 m. To minimize any potential stress on the zebrafish during the testing phase and to reduce experimental error, the outer walls of the tank were covered with black fabric. The tank was positioned on a level surface and filled with water from the zebrafish housing tank, with the water level reaching 5 cm below the upper edge of the test tank. This specific water level was chosen to enable the zebrafish to swim horizontally, thereby decreasing their vertical activity during the experiment. The duration of the test spanned four days. During the initial three days, each fish was allowed to adapt to the test tank for 5 min, twice daily. The time interval between these adaptation periods was 5 h. The training session was conducted 12 h after the final adaptation session and involved the exploration of two identical objects, which were yellow cubes (2.5 cm on by side), for 10 min. These cubes were positioned in two corners of the tank and were oriented parallel to each other. Following the training session, a one-hour retention period was implemented, during which one of the yellow cubes was replaced by a white one. Consequently, the yellow cube was deemed familiar (FO), while the blue cube was considered new (NO). In the subsequent test session, each zebrafish was permitted to explore the two cubes for 10 min. The assessment of the fish’s recognition memory was based on the percentage of preference. The fish’s preference (%) for one of the two cubes (calculated as the difference between the time spent exploring the novel cube versus the familiar cube) was determined using the following formula: time spent exploring the novel cube/(time spent exploring the familiar cube + time spent exploring the new cube) × 100. We also evaluated the total time of exploration of the NO and FO (s) by fish in the testing period.

#### 3.11.4. Biochemical Parameters

Immediately following the completion of the final behavioral assessment, the animals involved in the study were individually placed into glass containers containing ice-cold water maintained at a temperature range of 2–4 °C for a duration of 300–400 s. Following this, the animals were humanely euthanized via decapitation, adhering to established protocols as previously described. The entire brains were carefully extracted, weighed, and then transferred into 0.5 mL tubes. These samples were then stored at a low temperature of −20 °C until utilization in subsequent procedures.

The following day, the zebrafish brains from the same group were individually weighed (typically ranging from approximately 3 to 6 mg each) and subsequently homogenized in a phosphate extraction buffer consisting of 0.1 M potassium phosphate buffer with a pH of 7.4, supplemented with KCl at a concentration of 1.15%, at a ratio of 1:10. This homogenization process was conducted using a ball mill (Mikro-Dismembrator U; Sartorius, NY, USA). Following homogenization, the resulting suspension was centrifuged (for 15 min at a speed of 14,000 rpm).

The supernatant obtained was utilized for conducting biochemical assays, including assessments of AChE activity, employing the photometric method as detailed in Ellman’s paper [103]. CAT activity was measured using a simple colorimetric method originally described by Sinha [104] while SOD activity was determined using a protocol adapted from Winterbourne et al. [105]. GPX activity was assessed following the procedure detailed by Fukuzawa and Tokumura [106]. Additionally, the content of protein carbonyl groups was determined using a method originally described by Oliver et al. [107], and the level of MDA (lipid peroxidation) and carbonylated protein (protein oxidation) in the zebrafish’s brains were measured according to the protocols detailed by Ohkawa et al. [108] and Dalle-Donne et al. [109]. The protein content was quantified using the Bradford method [110].

### 3.12. Data Analysis

The results were expressed as the mean ± standard error of the mean (SEM). Group mean differences were assessed using a one-way analysis of variance (ANOVA), followed by a Tukey’s post hoc test, considering the treatment factor. The statistical significance was defined as *p* < 0.05. The GraphPad Prism 9.4 software (GraphPad Software, Inc., San Diego, CA, USA) was employed for all statistical analyses. Additionally, correlations between behavioral scores, enzyme activities, and lipid peroxidation were evaluated using the Pearson’s correlation coefficient (r).

## 4. Conclusions

The present study performed a probabilistic analysis of the primary compounds found in PGEO using in silico techniques. Furthermore, the investigation examined the impact of PGEO on memory processes in dementia using an animal model of zebrafish. The results show that most PGEO compounds possess the ability to cross the blood–brain barrier without causing hepatotoxicity. Despite the results obtained from in vivo experiments, the administration of PGEO did not cause noticeable changes in the behavior and antioxidant status of native zebrafish. However, it caused significant SCOP-induced amnesia, suggesting potential as a natural intervention for cognitive and behavioral disorders.

## Figures and Tables

**Table 1:** The chemical composition of *Citrus reticulata* essential oil leaves.

Identified Compounds	RI_exp_	RI_lit_	Content %
*p*-cymene	1015	1018	0.72 (±0.01)
Limonene	1019	1022	1.35 (±0.03)
*γ*-Terpinene	1050	1055	6.25 (±0.01)
Methyl-N-methyl anthranilate	1406	1402	89.93 (±0.08)
*β*-Caryophyllene	1411	1418	1.11 (±0.01)
Caryophyllene oxide	1576	1572	0.63 (±0.01)
Total identified			100

**Table 2:** Physicochemical properties of Scopolamine (SCOP), Galantamine (GAL), Methyl-N-methyl anthranilate, γ-Terpinene, Limonene, β-Caryophyllene, *p*-Cymene, and Caryophyllene oxide obtained using Pkcsm and SwissADME platforms.

Descriptor	SCOP	GAL	Methyl-*N*-Methyl Anthranilate	γ-Terpinene	Limonene	*β*-Caryophyllene	*p*-Cymene	Caryophyllene Oxide
Formula	C_16_H_22_O_9_	C_17_H_21_NO_3_	C_9_H_11_NO_2_	C_10_H_16_	C_10_H_16_	C_15_H_24_	C_10_H_14_	C_15_H_24_O
Molecular weight (g/mol)	303.35	287.359	165.19	136.23	136.23	204.35	134.22	220.35
Num. heavy atoms	22	21	12	10	10	15	10	16
Num. arom. heavy atoms	6	6	6	0	0	0	6	0
Fraction Csp3	0.59	0.53	0.22	0.60	0.60	0.73	0.40	0.87
Num. rotatable bonds	5	1	3	1	1	0	1	0
Num. H-bond acceptors	5	4	2	0	0	0	0	1
Num. H-bond donors	1	1	1	0	0	0	0	0
Molar Refractivity	83.48	84.05	47.03	47.12	47.12	68.78	45.99	68.27
TPSA	62.30 Å^2^	41.93 Å^2^	38.33 Å^2^	0.00 Å^2^	0.00 Å^2^	0.00 Å	0.00 Å	12.53 Å^2^

**Table 3:** The estimated pharmacokinetic parameters of drug-likeness and medicinal chemistry of the compounds analyzed in this study.

Druglikeness	SCOP	GAL	Methyl-N-Methyl Anthranilate	γ-Terpinene	Limonene	*β*-Caryophyllene	*p*-Cymene	Caryophyllene Oxide
Lipinski	Yes; 0 violation	Yes; 0 violation	Yes; 0 violation	Yes; 0 violation	Yes; 1 violation: MLOGP > 4.15	Yes; 0 violation	Yes; 0 violation	Yes; 0 violation
Ghose	Yes	Yes	Yes	No; 1 violation: MW < 160	No; 1 violation: MW < 160	Yes	No; 1 violation: MW < 160	No; 1 violation: MW < 160
Veber	Yes	Yes	Yes	Yes	Yes	Yes	Yes	Yes
Egan	Yes	Yes	Yes	Yes	Yes	Yes	Yes	Yes
Muegge	Yes	Yes	Yes/No; 1 violation: MW < 200	No; 2 violations: MW < 200, Heteroatoms < 2	No; 1 violation: Heteroatoms < 2	No; 1 violation: MW < 200	No; 2 violations: MW < 200, Heteroatoms < 2	No; 1 violation: MW < 200
Bioavailability Score	0.55	0.55	0.55	0.55	0.55	0.55	0.55	

**Table 4:** Prediction of permeability of intestinal absorption (human), skin permeability, VDss (human), BBB permeability, CNS permeability, CYP3A4 substrate, CYP1A2 inhibition, total clearance, renal OCT2 substrate, max. tolerated dose (human) and hepatotoxicity.

Property	Compound Model Name	SCOP	GAL	Methyl-N-Methyl Anthranilate	γ-Terpinene	Limonene	*β*-Caryophyllene	*p*-Cymene	Caryophyllene Oxide	Unit
Absorption	Intestinal absorption (human) (low < 30%, high > 30%)	72.626	94.994	93.334	96.219	95.301	95.898	95.561	94.547	Numeric (% Absorbed)
	Skin permeability (low logKp > −2.5, high logKp < −2.5)	−4.097	−3.75	−2.165	−1.489	−1.438	−1.721	−1.575	−1.017	Numeric (log Kp)
Distribution	VDss (human) (low log VDss < −0.15, high VDss > 0.45)	0.583	0.89	−0.15	0.412	0.412	0.396	−0.663	0.686	Numeric (log L/kg)
	Fraction unbound (human)	0.414	0.36	0.413	0.42	0.424	0.396	0.258	0.213	Numeric (Fu)
	BBB permeability (log BB > 0.3 cross BB, log BB < 0.1 do not cross BB)	−0.043	−0.081	−0.087	0.754	0.741	0.484	0.73	0.531	Numeric (log BB)
	CNS permeability (log PS > −2, penetrate CNS, log PS < −3 do not penetrate)	−3.031	−2.511	−1.785	−2.049	−2.029	−2.172	−2.172	−1.398	Numeric (log PS)
Metabolism	CYP3A4 substrate	Yes	Yes	No	No	No	No	No	No	Categorical (Yes/No)
	CYP1A2 inhibitor	No	No	No	No	No	No	No	Yes	Categorical (Yes/No)
Excretion	Total Clearance	1.096	0.991	0.75	0.217	0.217	0.213	1.088	0.239	Numeric (log ml/min/kg)
	Renal OCT2 substrate	No	Yes	No	No	No	No	No	No	Categorical (Yes/No)
Toxicity	Max. tolerated dose (human) (low < 0.447, high > 0.477)	−0.319	−0.423	0.769	0.756	0.63	0.77	0.271	0.858	Numeric (log mg/kg/day)
	Hepatotoxicity	No	Yes	No	No	No	No	No	No	Categorical (Yes/No)

The intestinal environment provides several key features that influence drugs.

**Table 5:** SMILE data for Brexpiprazole (BREX), Memantine (MEM), Donepezil (DON), Rivastigmine (RIV), and Galantamine (GAL).

Name	Smile	Chemical Structure
SCOP	CN1C2CC(CC1C3C2O3)OC(=O)C(CO)C4=CC=CC=C4	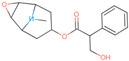
GAL	CN1CCC23C=CC(CC2OC4=C(C=CC(=C34)C1)OC)O	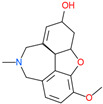
Methyl-N-methyl anthranilate	CNc1ccccc1C(=O)OC	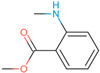
γ-Terpinene	CC1=CCC(C(C)C)=CC1	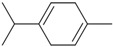
Limonene	C=C(C)C1CC=C(C)CC1	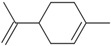
β-Caryophyllene	C=C1CCC=C(C)CCC2C1CC2(C)C	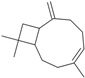
p-Cymene	Cc1ccc(C(C)C)cc1	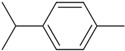
Caryophyllene oxide	C=C1CCC2OC2(C)CCC2C1CC2(C)C	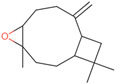

## Data Availability

Data are contained within the article.
